# Pre-injury dispensing of psychoactive prescription drugs in a ten years trauma population: a retrospective registry analysis

**DOI:** 10.1186/s13049-021-00939-6

**Published:** 2021-08-28

**Authors:** Henrik Andreas Torp, Svetlana Skurtveit, Nils Oddvar Skaga, Ingebjørg Gustavsen, Jon Michael Gran, Leiv Arne Rosseland

**Affiliations:** 1 Department of Pharmacology, Division of Laboratory Medicine, Oslo University Hospital, Oslo, Norway; 2 Department of Anaesthesiology, Division of Emergencies and Critical Care, Oslo University Hospital, Oslo, Norway; 3 Division of Critical Care, Institute of Clinical Medicine, University of Oslo, Oslo, Norway; 4 Norwegian Centre for Addiction Research, Institute of Clinical Medicine, University of Oslo, Oslo, Norway; 5 Department of Mental Disorders, Norwegian Institute of Public Health, Oslo, Norway; 6 Department of Research and Development, Division of Emergencies and Critical Care, Oslo University Hospital, Oslo, Norway; 7 Department of Biostatistics, Oslo Centre for Biostatistics and Epidemiology, Institute of Basal Medical Sciences, University of Oslo, Oslo, Norway

## Abstract

**Background:**

The use of psychoactive prescription drugs is associated with increased risk of traumatic injury, and has negative impact on clinical outcome in trauma patients. Previous studies have focused on specific drugs or subgroups of patients. Our aim was to examine the extent of psychoactive drug dispensing prior to injury in a comprehensive population of trauma patients.

**Methods:**

The Oslo University Hospital Trauma Registry provided data on all trauma patients admitted to the trauma centre between 2005 and 2014. We linked the data to Norwegian Prescription Database data from 2004. Opioids, benzodiazepines, z-hypnotics, gabapentinoids, and centrally acting sympathomimetics dispensed during the year before trauma of each patient were identified. We determined the pre-trauma annual prevalence of dispensing and mean annual cumulative defined daily doses (DDD) for each drug class, and compared results with corresponding figures in the general population, using standardised ratios. For each drug class, dispensing 14 days preceding trauma was analysed in patients sustaining severe injury and compared with patients sustaining non-severe injury.

**Results:**

12,713 patients (71% male) were included. Median age was 36 years. 4891 patients (38%) presented with severe injury (Injury Severity Score > 15). The ratio between annual prevalence of dispensed prescriptions for trauma patients and the general population, adjusted for age and sex, was 1.5 (95% confidence interval 1.4–1.6) for opioids, 2.1 (2.0–2.2) for benzodiazepines, 1.7 (1.6–1.8) for z-hypnotics, 1.9 (1.6–2.2) for gabapentinoids, and 1.9 (1.6–2.2) for centrally acting sympathomimetics. Compared with the general population, mean annual cumulative DDD of opioids and benzodiazepines dispensed to trauma patients were more than two and three times as high, respectively, in several age groups below 70 years. The prevalence of dispensing 14 days pre-trauma was higher in severely injured patients for opioids, benzodiazepines, and z-hypnotics compared with patients without severe injury.

**Conclusions:**

Our results support previous findings that the prevalence of psychoactive drug use is high among trauma patients. In terms of both frequency and amounts, the pre-injury dispensing of psychoactive drugs to trauma patients supersedes that of the general population, especially in younger patients.

## Background

Traumatic injury following accidents, violence, or other external exposures is an important cause of mortality and morbidity, particularly in adolescents and young adults. In 2018, Norwegians aged 15 to 29 years died more frequently from accidents than from disease [1]. It has been established that the use of psychoactive drugs, both prescription drugs and illicit drugs, is widespread in trauma patients [2, 3]. Studies have indicated that drug use before trauma is associated with poorer clinical outcome [4]. Knowledge of the extent of psychoactive drug use is therefore of vital importance in trauma care, both in treatment as well as in prevention of trauma.

Prescription opioid analgesics, benzodiazepine anxiolytics and hypnotics, and hypnotics like zopiclone and zolpidem (z-hypnotics) are widely used drugs that exert central nervous system (CNS) effects, causing sedation and impaired psychomotor functioning. These effects reduce attention and responsiveness, and impose an increased risk of traumatic injury. Indeed, opioids, benzodiazepines, and z-hypnotics have been identified as risk factors for road traffic accidents [5–7], falls [8–10], and occupational injury [11, 12]. Recently, the anticonvulsant and analgesic drugs gabapentin and pregabalin, collectively named gabapentinoids, have also attracted attention due to their misuse potential and increased risk of injury among their users [13, 14].

Amphetamine and amphetamine-like drugs used to treat attention deficit/hyperactivity disorder (ADHD) are increasingly prescribed in Norway [15]. Whether the use of these centrally acting sympathomimetics (CAS) modify the risk of injury is not entirely clear, although there is some evidence that it might lower the risk in ADHD patients when used appropriately [16, 17].

Previous research in the field has often been focused on particular causes, drugs, or age groups, and there is a lack of studies on drug use in trauma populations that are more comprehensive with respect to these factors.

Our study aimed at analysing prescriptions of several psychoactive drugs in trauma patients admitted to the largest regional trauma centre in Norway. Information on prescription drugs dispensed from pharmacies to patients before trauma reflects the use of these drugs in the trauma patient population. Specifically, our aims were

1. to determine the prevalence of dispensed opioids, benzodiazepines, z-hypnotics, gabapentinoids, and CAS in patients during the year before trauma, and compare it to the one-year prevalence in the general population,
2. to determine the dispensed amounts of drugs in patients during the year before trauma, and compare it to the general population, and
3. to compare patients with severe injury to patients with non-severe injury with respect to dispensing during the last 14 days before trauma.

## Methods

To study the dispensing of prescription drugs to trauma patients prior to injury, we linked data from the Oslo University Hospital Trauma Registry (OUH-TR) with data from the Norwegian Prescription Database (NorPD). Linkage was performed using each individual’s Norwegian national identity number.

### Oslo University Hospital Trauma Registry

Oslo University Hospital (OUH) is the major trauma hospital for more than 690,000 citizens in Oslo and the trauma referral centre for 3,000,000 people in the South-Eastern Norway Regional Health Authority (SENRHA) region. SENRHA is the largest of the four regional health authorities in Norway providing specialised health care. OUH admits approximately 1800 trauma patients per year. In addition to primary admissions, trauma patients from local hospitals are frequently transferred to OUH for advanced treatment. Trauma care services are allocated to Ullevål, which is one of the principal hospital facilities within OUH. The hospital established OUH-TR in 2000 for internal quality assurance purposes. The following patient categories are included in OUH-TR:

- All patients with recognised or suspected severe traumatic injury assessed by the trauma team upon admittance.
- All patients with penetrating injuries proximal to elbow or knee.
- Patients with Injury Severity Score (ISS) > 9 [18] or New Injury Severity Score (NISS) > 12 [19] not assessed by the trauma team.

The following categories are not included in OUH-TR (unless assessed by the trauma team):

- Patients with isolated fractures in one extremity, regardless of ISS or NISS.
- Patients with chronic subdural haematoma or isolated orbital floor fracture.
- Patients who sustained injury > 24 h prior to transfer from another hospital.

### The Norwegian Prescription Database

Since 2004, all pharmacies in Norway report detailed data on dispensed prescription drugs to NorPD [15]. Covering the entire population of Norway, the database is authorized by separate regulation in Norwegian legislation, and administered by the Norwegian Institute of Public Health. Information on drugs sold over the counter without prescription and drugs given to patients in hospital or other healthcare institutions is not included in the database. The Anatomical Therapeutic Chemical (ATC) classification is utilised for classifying dispensed drugs [20]. Amounts of drugs dispensed to patients are measured as defined daily doses (DDD).

### Retrieval, linkage, and coding of data

Data on all trauma patients from 2005 to 2014 were retrieved from OUH-TR. Patients included in OUH-TR who later turned out to have conditions not resulting from physical trauma (e.g. intoxications, cardiac arrest, epileptic seizures) were excluded from the dataset. Furthermore, patients that did not have a proper Norwegian national identity number, e.g. citizens of other countries not residing in Norway, were excluded. NorPD then retrieved data on all prescription drugs dispensed to patients during the year prior to each patient’s trauma, creating a data set of dispensed drugs from 2004 to 2014. Finally, the two data sets were pseudonymised, and all trauma dates and dispensing dates were replaced by independent time variables.

Anatomical injury was classified according to the Abbreviated Injury Scale 1990 – update 98 (AIS 98) [21]. Overall anatomical injury, i.e. ISS and NISS, were based on AIS 98 and calculated according to convention. ISS and NISS range is 1–75 where ISS/NISS 1 represents minor injury, ISS/NISS 75 represents lethal injury and ISS > 15 is defined as severe injury. Glasgow Coma Scale (GCS) score [22] was used for coding of level of consciousness on admission. Physiological derangement on admission was scored according to the Triage Revised Trauma Score (T-RTS) [23]. The T-RTS range 0–12 (12 is normal) is defined as the sum of the clinical category values of GCS score, systolic blood pressure, and respiratory rate on admission. Pre-injury co-morbidity was indexed according to the American Society of Anesthesiologists physical status classification (ASA) score [24]. ASA score 1 represents no disease and ASA score 4 severe disease that is a constant threat to life. Probability of survival (Ps) is presented by the Norwegian Prediction Model in Trauma II (NORMIT II) [25], based on well-founded predictors of trauma outcome; anatomic injury, physiological derangement, age and pre-injury comorbidity.

### Data analysis

The data sets were analysed using IBM SPSS Statistics for Windows, version 27.0 (IBM Corporation, Armonk, NY). Dispensed opioids (ATC code N02A), benzodiazepines (N05BA, N05CD, and N03AE01), z-hypnotics (N05CF), gabapentinoids (N03AX12 and N03AX16), and CAS (N06BA) were identified for individual patients. Dispensed drugs during the year prior to trauma were defined as any filled prescription from 365 days before trauma to the day before trauma. In this way, possible filled prescriptions on the day of trauma were omitted, since dispensing that day could have occurred after the patient was discharged from hospital care. In patients having been admitted more than once during the study period, we considered dispensed drugs prior to their first admission only.

To study differences across age and sex for each drug class, we divided the patients into nine ten-year age groups and split the groups according to female or male sex. We then used publicly available NorPD data [15] to determine the mean one-year prevalence of dispensing in the population of the SENRHA area for each drug class in each of the groups according to age and sex across the study period, i.e. 2004 to 2014. These prevalence figures were used to calculate expected dispensing in the patients of the OUH-TR data set, given the corresponding age- and sex-specific dispensing in the general population. The reason for choosing the SENRHA population prevalence was that a vast majority of the trauma patients in OUH-TR belong to this region. The ratio between observed and expected dispensing was then calculated for each drug class, along with the 95% confidence interval (CI) for each ratio [26]. For comparison, we chose prescription drugs without significant CNS effects that are used across all ages, selective β_2_ adrenoceptor agonists for inhalation (ATC code R03AC), and the third generation antihistamine desloratadine (R06AX27), and calculated the ratios in a similar way.

Mean annual cumulative DDD dispensed during the year preceding trauma was calculated for each drug class and age group, and compared with the mean annual cumulative DDD dispensed in the SENRHA population across the study period.

Dispensing of drugs during a period of fourteen days prior to trauma was studied in trauma patients sustaining severe traumatic injury (ISS > 15) and compared with trauma patients with non-severe traumatic injury (ISS < 15) or no injury at all.

## Results

### Study population characteristics

12,713 patients with 13,064 admissions were retrieved from OUH-TR, 8986 (71%) of which were men. 271 (2%) were admitted as trauma patients on two or more separate occasions during the study period. Basic characteristics of the study population are summarised in Table 1.

**Table 1:** Basic characteristics of the trauma patient study population

	Female	Male
Patients, *n* (% of total)	3727 (29)	8986 (71)
Age in years, median (range; IQR)	37 (0-103; 19–60)	36 (0–98; 22–52)
Scoring systems values, median (range; IQR)		
Pre-injury ASA	1 (1–4; 1–2)	1 (1–4; 1–2)
GCS score	15 (3–15; 14–15)	15 (3–15; 14–15)
T-RTS	12 (1–12; 12–12)	12 (1–12; 12–12)
ISS	10 (1–75; 4–20)	10 (1–75; 5–21)
NISS	12 (1–75; 4–27)	14 (1–75; 5–27)
Ps	0.995 (0.002–0.999; 0.967–0.999)	0.996 (0.001–0.999; 0.977–0.999)
Mechanism of injury, *n* (% within sex)		
Blunt	3569 (96)	8093 (90)
Penetrating	158 (4)	892 (10)
Unknown	0 (0)	1 (0)
Injury circumstances, *n* (% within sex)		
Transportation	1670 (45)	3647 (41)
Falls	1265 (34)	2718 (30)
Violence	183 (5)	1162 (13)
Sports and leisure	361 (10)	715 (8)
Occupational	25 (1)	558 (6)
Days of hospitalization, median (range; IQR)	3 (1–90; 2–7)	3 (1–105; 2–7)
ICU treatment, *n* (% within sex)	3128 (84)	7812 (87)
Days of ICU treatment, median (range; IQR)	2 (1–89; 1–3)	2 (1–105; 1–4)
Deaths within 30 days, *n* (% within sex)	225 (6)	482 (5)

12,713 trauma patients included in the Oslo University Hospital Trauma Registry between 2005 and 2014 comprised the study population. This table summarises key demographic and clinical data. IQR, interquartile range; ASA, American Society of Anesthesiologists physical status classification; GCS, Glasgow Coma Scale; T-RTS, Triage Revised Trauma Score; ISS, Injury Severity Score; NISS, New Injury Severity Score; Ps, Probability of survival according to Norwegian prediction Model in Trauma II (NORMIT II) score; ICU, intensive care unit

The traumatic injuries for which the patients were admitted to the trauma centre were most commonly associated with transportation incidents (42% of all patients). However, in the age groups 0–9 years, 70–79 years, and 80 years and above, injuries from falling were more common. Injuries associated with violence had their highest prevalence among male patients aged 20 to 29 years (25% of all injuries within the age group), whereas injuries sustained during sports or leisure activities were most prevalent in patients aged 10 to 19 years (27% of female and 21% of male patients within the age group). In male patients, occupational injuries were more frequent than sports and leisure injuries in all ten-year age groups between 30 and 69 years, ranging from 6 to 10% and 2 to 7%, respectively.

4891 patients (38%) presented with severe trauma (ISS > 15).

### Dispensed prescription drugs

During the year before trauma, 1853 patients (15%) filled at least one prescription for opioids, 1452 (11%) for benzodiazepines, 1362 (11%) for z-hypnotics, 153 (1%) for gabapentinoids, and 159 (1%) for CAS. 3297 patients (26%) filled at least one prescription for any drug within the five aforementioned drug classes. Among patients who filled prescriptions for opioids, 861 (46%) filled only one prescription. For benzodiazepines, z-hypnotics, gabapentinoids, and CAS, the corresponding numbers were 359 (25%), 401 (29%), 40 (26%), and 20 (13%), respectively. Table 2 provides further details and accounts for sex differences.

**Table 2:** Dispensed drugs one year pre-injury

	Patients, *n* (%)		Filled prescriptions per patient, median (IQR)		Patients having filled only one prescription, *n* (%)	
	Female	Male	Female	Male	Female	Male
Opioids	659 (18)	1194 (13)	2 (1–6)	2 (1–4)	270 (41)	591 (49)
Benzodiazepines	554 (15)	898 (10)	4 (1–12)	5 (1–13)	153 (28)	206 (23)
Z-hypnotics	614 (16)	748 (8)	3 (1–7)	3 (1–6)	167 (27)	234 (31)
Gabapentinoids	60 (2)	93 (1)	3.5 (2–8)	4 (1–8)	13 (22)	27 (29)
Centrally acting sympathomimetics	44 (1)	115 (1)	4.5 (3-7.75)	5 (3–9)	4 (9)	16 (14)

Psychoactive prescription drugs dispensed to trauma patients one year pre-injury, according to drug class. IQR, interquartile range

The ratio between observed dispensing and expected dispensing was 1.5 (95% CI 1.4–1.6) for opioids, 2.1 (95% CI 2.0-2.2) for benzodiazepines, 1.7 (95% CI 1.6–1.8) for z-hypnotics, 1.9 (95% CI 1.6–2.2) for gabapentinoids, and 1.9 (95% CI 1.6–2.2) for CAS. In comparison, the ratio was 1.2 (95% CI 1.1–1.2) for selective β_2_ adrenoceptor agonists, and 0.8 (95% CI 0.7–0.9) for desloratadine. Differences between women and men are stated in Table 3.

**Table 3:** Expected and observed drug dispensing in trauma patients

	Female			Male		
	Expected	Observed	Ratio observed: expected (95% CI)	Expected	Observed	Ratio observed: expected (95% CI)
Opioids	432	659	1.5 (1.4–1.6)	803	1194	1.5 (1.4–1.6)
Benzodiazepines	312	554	1.8 (1.6–1.9)	394	898	2.3 (2.1–2.4)
Z-hypnotics	371	614	1.7 (1.5–1.8)	442	748	1.7 (1.6–1.8)
Gabapentinoids	31	60	1.9 (1.4–2.4)	49	93	1.9 (1.5–2.3)
Centrally acting sympathomimetics	16	44	2.8 (1.9–3.6)	67	115	1.7 (1.4-2.0)
Selective β_2_ adrenoceptor agonists	206	249	1.2 (1.1–1.4)	396	449	1.1 (1.0-1.2)
Desloratadine	103	91	0.9 (0.7–1.1)	197	148	0.8 (0.6–0.9)

Expected and observed drug dispensing in trauma patients one year pre-injury. Ratios are stated with 95% confidence intervals (CI). Expected values were calculated based on data from the Norwegian Prescription Database

**Fig. 1:**
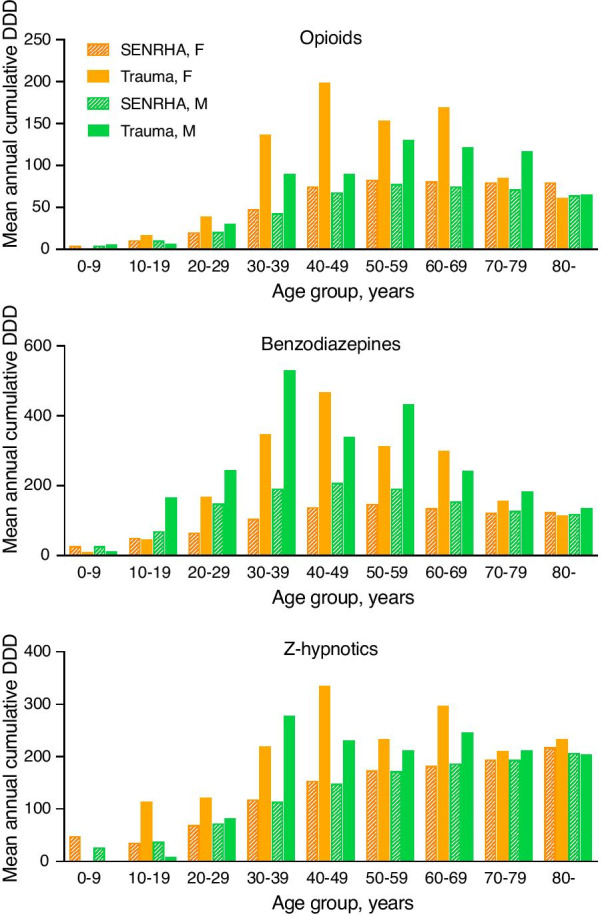
Mean annual cumulative defined daily doses (DDD) of opioids, benzodiazepines, and z-hypnotics in the study population of trauma patients and in the South-Eastern Norway Regional Health Authority (SENRHA) population, according to sex (F: female; M: male) and age group. Please observe that the scale of the vertical axis varies between the different charts

Figure 1 presents mean annual cumulative DDD of opioids, benzodiazepines, and z-hypnotics dispensed in trauma patients during the year before trauma and in the SENRHA population during 2009. For opioids, the differences were particularly noticeable in young and middle-aged female trauma patients (up to 2.8 times higher amount in female patients aged 30 to 39 years compared with the SENRHA population). For benzodiazepines, differences were more than three-fold in females 30 to 39 and 40 to 49 years old. In males, the highest mean annual cumulative benzodiazepine DDD was seen in patients aged 30 to 39 years, 2.8 times higher compared with the SENRHA population. A similar pattern was observed for z-hypnotics, although less pronounced. When comparing mean annual cumulative DDD of selective β_2_ adrenoceptor agonists, we found that the study population of trauma patients followed the SENRHA population more closely.

### Dispensed prescription drugs in severely injured patients

The proportions of patients having filled prescriptions for opioids, benzodiazepines, and z-hypnotics during the fourteen days prior to trauma were higher in patients sustaining severe injury compared with patients sustaining non-severe injury, or for whom severity was unknown. For gabapentinoids and CAS, the proportions were lower among severely injured patients. Details are provided in Table 4.

**Table 4:** Dispensed drugs fourteen days prior to trauma in patients with severe and non-severe injury

	ISS > 15		ISS < 15 or unknown	
	*n*	%	*n*	%
All patients	4891	100	7822	100
Opioids	134	2.7	151	1.9
Benzodiazepines	200	4.1	235	3.0
Z-hypnotics	133	2.7	155	2.0
Gabapentinoids	14	0.3	28	0.4
Centrally acting sympathomimetics	6	0.1	22	0.3

Trauma patients with severe injury, i.e. Injury Severity Score (ISS) > 15, and patients with non-severe injury (ISS < 15) or unknown severity, were compared with respect to the proportion of patients who had been dispensed drugs during the last fourteen days preceding trauma.

## Discussion

The present study analysed dispensing of psychoactive prescription drugs in trauma patients of all ages admitted to Oslo University Hospital from 2005 to 2014. The results demonstrate that pre-injury dispensing of opioids, benzodiazepines, z-hypnotics, gabapentinoids, and CAS is more widespread among trauma patients than in the general population, and that dispensed doses of these drugs were considerably larger in younger trauma patients in particular. In contrast, similar differences in dispensing of selective β_2_ adrenoceptor agonists and desloratadine were absent. Additionally, we found that dispensing of opioids, benzodiazepines, and z-hypnotics during the last fourteen days prior to trauma was more frequent in patients with severe injury than in patients with non-severe injury.

The high prevalence of dispensing could be related to pre-existing morbidity in the trauma patient population, like pain conditions and psychiatric disorders. Indeed, studies from the United States have indicated that almost half of all trauma patients suffer from psychiatric conditions, including substance use disorder [27]. We found that the prevalence of opioid, benzodiazepine, z-hypnotic, and gabapentinoid dispensing was higher in female trauma patients compared with male trauma patients. This is consistent with the situation in the Norwegian general population as well [28]. The increased amounts of dispensed drugs found in our study were also more prominent in female patients. However, the most important observation in this context was that the large differences in the amounts of dispensed drugs were primarily confined to young and middle-aged trauma patients when compared with the general population. Our observation might be explained by age-related variations in prevalence of pre-injury morbidity among trauma patients: In patients aged 18 to 64 years, substance use disorder is present in almost 50%, whereas it is less common in older patients [27].

Many previous studies have demonstrated an increased risk of injury associated with psychoactive drug use [29–31]. In the trauma population we studied, it is likely that some of the patients were under the influence of drugs when injured. On the other hand, any dispensing during the entire year before does not necessarily implicate drug use at the time of trauma. However, studying dispensing during the last fourteen days before trauma might give a stronger indication of ongoing drug use. The clear differences between patients sustaining severe injury and patients sustaining non-severe injury point towards a possible involvement of drug effects. In patients with ADHD, there is an underlying risk of injury due to impaired impulse control and risk-seeking behaviour [32]. Our analysis show that in patients sustaining non-severe injury, the proportion having been dispensed drugs for treatment of ADHD is larger than in patients sustaining severe injury. The difference between the groups is minor, but the results support previous findings that pharmacological treatment of ADHD reduces the risk of injury [33, 34].

Comparing our results to results from other studies is difficult due to methodological differences. Cannon and co-workers [3] analysed a sample of 1700 trauma patients retrospectively and found that 20% were on prescription opioids, benzodiazepines, or both, according to their medication reconciliation forms. This is comparable to the 26% found in our study, considering we included additional drug classes and included all dispensing during one year. Furthermore, they found that a higher proportion of female trauma patients were on these prescription drugs compared with male patients, which is also consistent with our findings. Some studies have analysed blood samples from patients admitted to hospitals for injuries. In the study by Bogstrand et al., medicinal drugs were detected in the blood samples of 21% of injured patients above 18 years of age (*n* = 1272) [35]. The drugs that were detected were in large part benzodiazepines, z-hypnotics, and opioids. The prevalence of drug presence in blood samples is not far from the one-year pre-injury dispensing prevalence we found. This demonstrates that dispensing one year prior to trauma might indicate actual drug use at the time of trauma on a population level.

### Strengths and limitations

The large sample size allows us to alleviate uncertainty in our results, and increases the generalizability of the study. In addition, we analysed data from a population that was comprehensive, which also contributes to the generalizability. On the other hand, we might miss subtle but yet important variation between subgroups when regarding such a comprehensive population as a whole.

High-quality registry data have a high degree of completeness, and eliminate the risk of recall bias and information bias. One disadvantage of using data from prescription databases is the possibility that dispensed drugs are not used by the patient, and thus dispensing does not necessarily reflect drug use, as discussed previously. However, studies have shown that there is a high degree of agreement between prescription data and self-reported use of psychoactive drugs [36, 37].

Alcohol use and illicit drug use are frequently associated with trauma [2, 35]. As the provided registry data did not include information on such use, this study setting did not allow us to examine concomitant use of prescription drugs and alcohol or illicit drugs.

By standardising to age and sex, we were able to compare trauma population data with general population data. Still, the use of matched controls would have offered the ability to compare data more accurately. For privacy reasons, neither the patient’s municipality of residence nor year of trauma were disclosed in the OUH-TR data set. Therefore, it was impossible to omit trauma patients residing within the SENRHA area from the SENRHA population used to calculate prevalence, and these patients are included in both populations. This might have led to an underestimation of differences between the trauma population and the general population.

### Clinical relevance

Knowledge about the scope of drug use in trauma patients is of great importance to anyone providing health care to patients with traumatic injuries, in both pre- and in-hospital settings. Unrecognised and untreated drug intoxications impose a serious risk to any trauma patient, and insufficient attention to previous drug use may cause withdrawal symptoms and impair treatment and recovery.

In terms of prevention, prescribers should be aware of the high prevalence of pre-injury psychoactive drug dispensing among trauma patients, and avoid long-term prescribing, inappropriately high dosing, and combinations of drugs with sedative properties. Patients should be informed of the adverse effects and the risks associated with these drugs, and be discouraged from exceeding prescribed dosing and combining psychoactive drugs and alcohol.

Traumatic injury represents a significant burden, not only to the individual, but also to society. As it is a major cause of mortality and morbidity in young people, it contributes to loss of manpower and high health care and social care expenditures in an otherwise healthy part of the population. Therefore, authorities should consider targeting the prescription of psychoactive drugs when implementing strategies to prevent accidental trauma.

## Conclusions

- In trauma patients, the prevalence of psychoactive prescription drug dispensing one year pre-trauma is higher compared with the one-year prevalence in the general population. The dispensed doses are also higher in trauma patients. This applies to younger and middle-aged patients in particular.
- Severely injured patients have more often been dispensed psychoactive drugs 14 days pre-trauma than non-severely injured patients.
- Our findings are important to pre- and in-hospital trauma care providers, and to prescribers of psychoactive drugs.

## Data Availability

Access to study data is restricted to the researchers as specified in the approvals given by the Regional Committee for Medical and Health Research Ethics and the hospital’s Data Protection Officer. However, access may be granted following application. Requests should be made to Leiv Arne Rosseland (l.a.rosseland@medisin.uio.no).
